# Suppression of Face Perception during Saccadic Eye Movements

**DOI:** 10.1155/2014/384510

**Published:** 2014-04-24

**Authors:** Mehrdad Seirafi, Peter De Weerd, Beatrice de Gelder

**Affiliations:** ^1^Cognitive and Affective Neuroscience Laboratory, Tilburg University, 5037 AB Tilburg, The Netherlands; ^2^Department of Cognitive Neuroscience, Faculty of Psychology and Neuroscience, Maastricht University, 6229 EV Maastricht, The Netherlands; ^3^Donders Institute for Brain, Cognition and Behaviour, Radboud University, 6500 GL Nijmegen, The Netherlands

## Abstract

Lack of awareness of a stimulus briefly presented during saccadic eye movement is known as saccadic omission. Studying the reduced visibility of visual stimuli around the time of saccade—known as saccadic suppression—is a key step to investigate saccadic omission. To date, almost all studies have been focused on the reduced visibility of simple stimuli such as flashes and bars. The extension of the results from simple stimuli to more complex objects has been neglected. In two experimental tasks, we measured the subjective and objective awareness of a briefly presented face stimuli during saccadic eye movement. In the first task, we measured the subjective awareness of the visual stimuli and showed that in most of the trials there is no conscious awareness of the faces. In the second task, we measured objective sensitivity in a two-alternative forced choice (2AFC) face detection task, which demonstrated chance-level performance. Here, we provide the first evidence of complete suppression of complex visual stimuli during the saccadic eye movement.

## 1. Introduction

We typically make hundreds of saccadic eye movements every minute [1, 2], resulting in a continuous shift of the visual world on the retina. Answering the question of how stable vision is achieved during saccade has been one of the major problems in unifying models for vision.

One of the key approaches in addressing this question is to study the dynamics of visual sensitivity in a time window around the saccadic eye movement. Several lines of research have revealed a dramatic deterioration of visual sensitivity at the time of saccade, known as saccadic suppression [3–6]. However, there is no general consensus about the domain and the underlying mechanisms of such sensitivity loss.

The source of sensitivity loss during saccade has been attributed to two different streams: top-down and bottom-up. In the top-down account, an active extraretinal suppression process is proposed [7] which targets only the magnocellular pathway [8] and does not affect the equiluminant visual stimuli [9]. On the other hand, the bottom-up account postulates a simple passive process of retinal motion smear as a source of suppression during saccade [10, 11]. In this account, the presence of high contrast spatial structure before and after the saccade introduces the masking of the blurred perisaccadic retinal image [12, 13].

One of the questions regarding saccadic suppression is its possible effect in processing more complex and more biologically significant visual objects. The majority of saccadic eye movement studies in the past century have generally employed simple stimuli such as bars, dots, and gratings with different spatial frequencies and small window sizes that affected luminance instead of pattern detection. Some recent studies employing modern gaze-contingent paradigms have been focused on vision around the time of freely made eye movements in natural dynamic scenes [14]. However, such paradigms are not able to dissociate the sensitivity change during saccade from the forward and backward masking effects introduced by the pre- and postsaccadic retinal images. Furthermore, none of these experiments has been focused on measuring the sensitivity loss during saccade for object detection or object recognition tasks.

One of the challenges of dealing with more realistic objects is how to measure the visual sensitivity in the object detection/categorization tasks. There is an ongoing debate about whether the objective or the subjective measure is appropriate for this purpose. Subjective measures are based on a subject's report about the task-relevant attribute (e.g., object category) of the stimulus. Objective measures rely on the participant's performance in a certain task regardless of the participant's conscious awareness of the task-relevant attribute. Consequently, to fill in this theoretical gap, a new approach has emerged in recent years which takes into account both subjective and objective measures [15–17].

In the present study, we investigated the levels of saccadic suppression for the category of complex visual objects. The goal was to see whether a salient visual stimulus could still be rendered fully invisible if presented during saccadic eye movement. This was done by measuring the sensitivity loss during saccade for face stimuli using two separate tasks. In the first task, we recorded the subjective rating of the stimuli that were presented during saccade. In the second task, we measured the participants' objective face detection performance.

## 2. Methods and Materials

### 2.1. Participants

We recruited 14 participants (11 females, *M*_age_: 24, age range: 18–32) from Maastricht University through local advertisements for this study. The study was conducted in accordance with university ethics committee requirements. The subjects gave informed consent to participate and were rewarded with 7.5 Euro vouchers. All participants were right-handed, with normal or corrected-to-normal vision and naive as to the purpose of the experiment. Two participants were excluded from the analysis due to the fact that they always pressed the same button to all stimuli in all conditions during the face detection task (see below).

### 2.2. Stimuli and Procedure

#### 2.2.1. Physical Setup

Movements of the right eye were measured using an EyeLink 1000 Desktop Mount (SR Research, Osgoode, ON, Canada) with an average spatial resolution of 0.25- to 0.5-, sampling at 1 kHz. Manual responses were recorded via a standard keyboard. The real-time connection between the eye tracker and the stimulus presentation control as well as the response collection was implemented in E-Prime 2.0 Professional software (Psychological Software Tools, Inc., Pittsburgh, PA).

#### 2.2.2. Image Preprocessing

Original face images were color images of two males and two females, each displaying the emotional conditions of happy, fearful, and neutral. These were selected from a subset of the Karolinska Directed Emotional Faces (KDEF) [18], previously validated to be correctly (>90%) categorized as happy, fearful, and neutral in a pilot study.

The stimuli were preprocessed as follows: first, all the original images were cropped and resized to fit into a rectangle matching eye position by Adobe Photoshop CS6 (Adobe; http://adobe.com/). Then, average pixel values for each image were shifted to 128 in Matlab 2012a (Mathworks; http://www.mathworks.com/). Scrambled faces (*scrambles*) were generated by randomizing the phases of Fourier transform of the preprocessed face stimuli while keeping the Fourier power constant. The phase shuffling is quantified by a phase coherence index, in which 0 means that all phase information has been randomized, preserving the overall distribution, and in which 100 means that all phase information is intact [19–21]. The main advantage of this technique is keeping the significant components for low-level vision (such as average luminance and Fourier power distribution) constant. The scrambles used in this experiment were generated at zero phase coherence.

#### 2.2.3. Procedure and Stimuli

Before the beginning of the first block, participants were familiarized with the experimental procedure using a short run of the subjective rating task (see below) consisting of 4–8 trials. After the practice phase, we showed them two target stimulus examples of the two experimental conditions in order to acquaint them with the stimuli of the scramble condition. Therefore, they were completely aware of possible face and scramble conditions.

**Figure 1:**
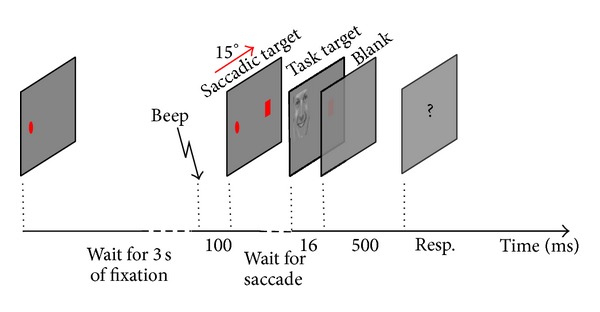
Schematic view of one trial: after 3 seconds, an auditory cue initiates the saccade followed by visual saccadic target. As soon as the saccade is detected, one visual stimulus (face or scramble) is presented, followed by a blank screen. Next, the task-relevant question is displayed on the screen.

A trial proceeded as follows. A red circle was displayed on horizontal median, 1/4 of the screen to the left horizontally on a gray background (Figure 1). After 3 seconds of fixation on the circle (diameter = 0.5°) as measured by the eye tracker, a beep cued the participants to make a saccade. The auditory cue was accompanied with a* saccadic target* indicating where the end-location of the saccade should be. The saccadic target was a small rectangle, 15° to the right of the fixation on the horizontal meridian. As soon as the participant initiated the saccade, a* task target* (width = 8.5°) was displayed on top of the initial fixation point (*saccadic origin*) for 16.7 ms. Next, a blank grey screen was shown for 500 ms, followed by the response screen including task-relevant question (see below).

#### 2.2.4. Design and Tasks

The participants performed two tasks in four separate experimental blocks: the first and the last blocks were the subjective rating tasks; the second and the third blocks were the face detection task.

*Task 1: Subjective Rating*. In the subjective rating task, the participants reported what they saw during the saccade. They were instructed to choose “nothing,” “something,” or “everything” by pressing the corresponding arrow keys on the keyboard: (a)* left arrow key* for “nothing,” when they did not perceive any target stimulus or its background flashing on the screen during the saccade; (b)* down arrow key* for “something,” when they saw a bright rectangle flashing on the screen but did not see anything inside it; (c)* right arrow key* for “everything,” if they saw the target stimulus which could have been a face or a noisy grey texture. In addition, the participants were always asked if they had perceived something other than the three alternatives, to be sure that our options were inclusive of all the possible choices. Each experimental block in the subjective rating task consisted of 24 trials. The scramble and each of the three emotional face conditions (neutral, happy, and fearful) had the same proportion.

*Task 2: Face Detection*. In the face detection task, the procedure was similar to the subjective rating task except that the participants were asked to report whether they perceived the stimuli as a* face* or as a* scramble*.* Left* and* right* button presses corresponded, respectively, to face and scramble responses. Each experimental block in the face detection task consisted of 32 trials. In half of the trials, face stimuli were presented (with the same proportion of all three emotional conditions), and in the other half, scramble stimuli were presented.

### 2.3. Data Analysis

#### 2.3.1. Signal Detection Theory

The sensitivity to the signal was estimated by calculating the* d*-prime (*d*′). The* d*′ is a measure of the distance between the signal and the noise distribution means in standard deviation units [22]. A* d*′ of 0 means that the participants are not able to discriminate the faces from scrambles in face detection task. The* d*′ was calculated as

(1) $$\begin{array}{c} d^{\prime }=\Phi^{-1}\left(H\right)-\Phi^{-1}\left(\text{FA}\right), \end{array}$$

where *H* is the hit rate (proportion of correctly responded trials with face stimuli) and FA is the false alarm rate (proportion of correctly responded trials with scramble stimuli). The function Φ^−1^ converts the rates into* z*-scores.

#### 2.3.2. Gaze Analysis and Saccade Detection

The saccades were detected based on the commonly used saccadic threshold for the velocity of eye movement based on weighted sum of four samples as follows:

(2) $$\begin{array}{c} V^{2}+{\text{V}}^{\prime 2}>v\;{\left(\text{thrs}\right)}^{2}, \end{array}$$

where *V* and *V*′ are components of pupil velocity on horizontal and vertical axes, respectively, and *v*  (thrs) is set to 40 degrees/second. Velocity for each sample was calculated by a weighted sum of four samples as follows:

(3) $$\begin{array}{c} V_{s[n]}\;=\frac{\left(1000\;\left(x_{\left[n+2\right]}+x_{\left[n+1\right]}-x_{\left[n-1\right]}-x_{\left[n-2\right]}\right)\right)}{\left(6\text{PP}{\text{D}}_{\left[n\right]}\right)}, \end{array}$$

in which the units are in true degree per second and PPD is the corresponding resolution for each sample. In order to ensure that only the correct horizontal saccades are detected, an additional constraint was set to exclude the saccades diverging more than 5 degrees from the horizontal axis.

## 3. Results

We first preprocessed the data by removing the trials without response and the trials where no saccade was detected within a time window of 10 seconds (*≈*5 percent of the whole trials). Then, we checked for any significant difference between different emotional conditions (happy, fearful, and neutral) in any of the two tasks by pooling the data from all participants. For the subjective rating task, we tested if there were any significant differences between the proportions of responses to the three emotional conditions. The Pearson Chi-square nonparametric test showed no significant difference between the proportions of responses to the three emotional conditions (*χ* ^2^(4, *N* = 401) = 2.77,   *P* = 0.60). Afterwards, we tested if the proportion of correct responses to the three different emotional conditions differed in the face detection task. For this, we applied analysis of variance (ANOVA) with the proportion of correct response as the dependent variable and emotion as the independent variable. The average proportion of correct responses for the three emotions (*M*_Fear_ = 0.43; *M*_Happy_ = 0.48; *M*_Neutral_ = 0.42) did not show a significant difference (*F*  (2,365) = 0.64; *P* = 0.52).

### 3.1. Subjective Rating Task

**Figure 2:**
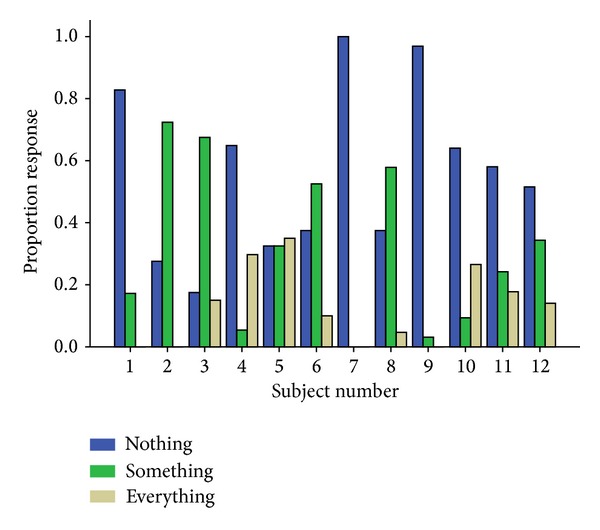
Proportion response in subjective rating task for every individual participant.

Figure 2 shows the proportion of responses to each of the three options in the subjective rating task. The results showed that none of the participants had conscious awareness of the stimulus category in most of the trials (“nothing” and “something” conditions).

Next, we analyzed the subjective measure of conscious perception of the stimuli in the subjective rating task averaged across the participants to see the extent of stimulus visibility during the saccade.

**Figure 3:**
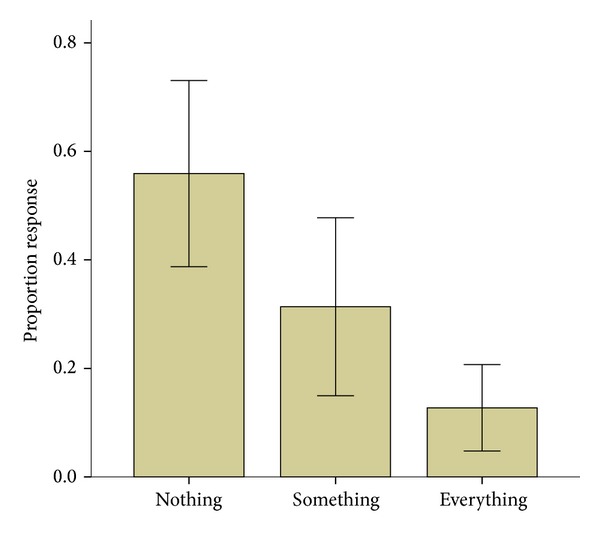
Proportion response to each of the three alternatives averaged across participants. The error bars represent 95% confidence interval.

As depicted in Figure 3, the results showed that in only a small portion of all trials (*≈*11%) the participants could consciously perceive the presented stimuli during a saccade, and in about one-third of the trials, the participants could only perceive a flashing square (the size of the face frame). In the remaining trials (*≈*56%), the participants perceived absolutely nothing during the saccade. It is noteworthy that, because of the dependence of the variables (proportion responses), analysis of variance (ANOVA) was not suitable; this might also have caused inflated significance of the correlation analysis. Thus, we compared the proportions of responses to the three response options with a nonparametric test. The Friedman test revealed a significant difference between the three options (*χ* ^2^(2,   *N* = 12) = 8.65, *P* = 0.013). Then, we compared all the pairs using a nonparametric test of Wilcoxon signed ranks. The results showed a nonsignificant difference between the “something” and “nothing” conditions (*P* > 0.1). The difference between “everything” and “something” was marginally significant (*Z* = −1.87; *P* = 0.062), and the difference between “everything” and “nothing” was significant (*Z* = −2.94; *P* = 0.003).

### 3.2. Face Detection Task

**Figure 4:**
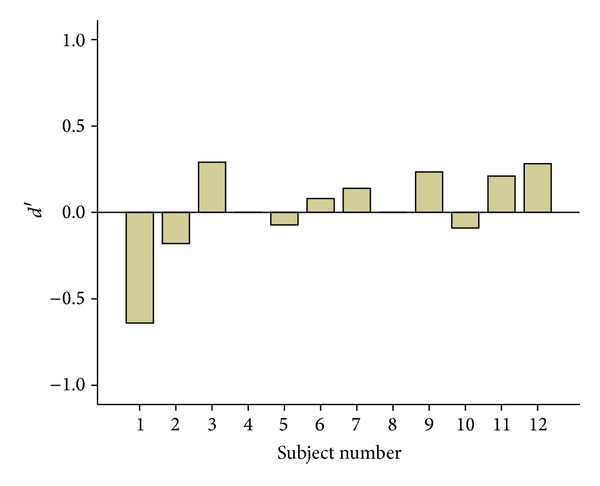
Sensitivity of individual participants in the face detection task.

First, we calculated* d*′ as a measure of sensitivity for each participant (see Section 2). Four participants exhibited below-chance behavior (negative* d*′) in the face detection task (Figure 4). A negative* d*′ at subject level can generally be related to either mislabeling the response options by the subject or a case of sampling error similar to the most subliminal studies [16, 23]. The former is quite unlikely in our experimental setup due to presentation of response options on the screen after every single trial; hence, the latter is most likely the case in this experiment. In group analysis, resetting negative* d*′s to zero value or simply omitting them from the analysis might lead to an inflated estimate of group* d*′ [24, 25]; thus, we kept them for the main analysis. Nevertheless, a post hoc analysis excluding only the highest negative* d*′ (subject 1) exhibited no difference in the significance of the statistical results (below).

Then, we compared the* d*′ with the baseline (*d*′ = 0) using Student's* t*-test across all of the participants. The average* d*′ for face detection was 0.02 ± 0.075. The results show that face detection is not significantly different from the baseline (*t*  (11) = 0.285; *P* = 0.78). We further analyzed the data using bootstrap for a one-sample* t*-test with 1000 iterations. The results were consistent with the previous test showing the mean difference of 0.02 (bias: 0.002; standard error: 0.07; *P* = 0.82).

### 3.3. Comparison of the Two Tasks

We further compared the results from the two tasks to examine the possible relationship of the proportion of responses of subjective rating from the first task and face detection sensitivity in the second task. For this, we calculated the Pearson correlation of each of the three response options in the subjective rating task with the* d*′ calculated from the second task across the participants. The correlation of face detection sensitivity to “everything,” “something,” and “nothing” responses was 0.132, 0.056, and −0.11, respectively. None of the correlations reached the significance level (*P* > 0.35).

## 4. Discussion

During saccadic eye movements, stable vision is maintained by the reduction of visual sensitivity. Here, we tested the extent of this reduction for complex stimuli that were briefly presented only during the saccadic eye movements. First, we showed that when a complex visual object is displayed during a saccade, the participants could not self-report its presence in 90% of trials (subjective rating task). Then, we used sensitivity analysis as an objective measure in a 2AFC face detection task. The results indicated near-zero discrimination sensitivity for faces versus scrambles. Finally, we examined the relationship between subjective rates from the first task and the face detection sensitivities from the second task across the participants. The results showed that none of the three rating conditions was significantly connected to the face detection performance. This suggests at least some degree of independence between the subjective and objective measures.

The results from the face detection task show that there is no awareness of the face even with objective measures during saccadic eye movement. It has been repeatedly demonstrated that the mere subjective indication of “not seeing” the visual stimuli does not exclude the fact that certain aspects of the stimulus cannot still be processed and revealed by a 2AFC task (for a review see [17]). Thus, the combination of the two tasks is necessary. However, the validity of the “seen” trials was not directly confirmed since the two tasks were run in different experimental blocks. It is important to validate these results with simultaneous responses to both objective and subjective tasks in every trial.

In the current theoretical framework of object detection, there is no consensus on how the conscious awareness is defined precisely. The main debate is whether the subjective or the objective measure is suitable to measure conscious awareness. However, a third approach has emerged in the past decade arguing that both accounts are important and each one is actually representing one distinct network [15–17]. By introducing the two levels of “unseen” conditions in this experiment, we aimed to examine the connection of those two subjective levels to subliminal face detection performance. However, due to the chance-level performance in the face detection task and lack of simultaneous measure of the two tasks, we were unlikely to find a strong correlation that is only based on correlation across the participants. The absence of such strong correlation suggests that the two subjective and objective measures are independent to some extent. This is consistent with the previous research demonstrating the divergence of the two measures in certain conditions and reiterates the necessity of running both tasks in similar research [16].

This experiment is relevant to the context of unconscious and subliminal perception from the methodological point of view. We showed that saccadic suppression is a powerful tool to mask complex visual stimuli completely. Masking has been a very important tool for studying the basic stages of visual processing. The results of this experiment show that saccadic eye movement can strongly disrupt the perception of the complex visual stimuli even if they are as salient as faces. This is interesting as rendering face stimuli invisible with traditional masking techniques is a difficult task particularly when they are presented at normal contrast, binocularly, and with large sizes [26, 27]. Furthermore, in forward and backward masking paradigms, both mask and target need to be displayed. To avoid the assumptions made about the interaction between the target and mask, saccadic suppression could be a useful alternative. Moreover, saccadic suppression does not need to include assumptions about the interaction of the different stimuli presented to each eye as in binocular rivalry where different stimuli are presented to different eyes and the percept of one eye suppresses the percept of the other eye. Hence, we believe that saccadic suppression can be employed as a complementary technique in a broad range of face/object detection and discrimination of experimental designs where the visibility of the target stimulus needs to be reduced.

The current study can also be relevant to the recent research revealing that certain properties of the “unseen” perisaccadic stimuli can still be processed unconsciously and influence the perception of the following stimuli. Recently, Watson and Krekelberg showed that the unconscious processing of an oriented bar that is presented during saccade can alter the perception of the upcoming stimulus at the end of saccade [28]. Moreover, there is an increasing body of evidence that the suppressed face stimuli can still be processed unconsciously. Other studies have demonstrated that unperceived objects can still activate cortical [29] and subcortical structures [30] under certain conditions. The results from the current study show that face detection is completely suppressed during saccade. Hence, it would be interesting to investigate if any subliminal perception can take place under these conditions. Future behavioral and imaging studies can address the possibility of such subliminal effects in the context of complex object perception. A remaining question is which underlying factors are involved in different ratings within and across the participants. For example, the timing of the stimulus presentation within the perisaccadic interval and the speed of saccade at the time of stimulus presentation can be two relevant factors for both subjective rating and face detection tasks.

The present work is also related methodologically to the studies using “change blindness” during saccadic eye movement [31]. Change blindness is the inability to detect the changes to an object or scene under certain conditions. The most relevant example, which also used complex objects, is research aiming to examine the rigidity of position-invariant object recognition [32]. In that study, the authors engineered a situation where the participants were instructed to saccade to the complex objects that were displayed peripherally. During the saccade, the identities of the peripheral objects were altered. In line with the present study, the authors first demonstrated that the participants were unaware of the identity change happening to the complex artificial target objects during saccade. Implementing this technique allowed them to construe a virtual environment, with different visual statistics, where the associations of the peripheral and the foveal representations of certain object identities were broken. They showed that the participants who were trained in such a “position-variant” environment would lose the position-invariant object recognition drastically for the trained objects. However, that study did not disentangle the masking effect of the presaccadic stimulus on the postsaccadic test stimulus, since they both were presented at the same position of saccadic landing. The other methodological difference was that they used artificial objects unlike the current study.

Examining the visual system under naturalistic conditions has been one of the main goals of this work. In the past decade, the extension of the findings from simple stimuli to the real-life vision has been debated in the basic vision research [33]. The main criticism of the opponents using simple stimuli is that the visual system has evolved and developed to optimally analyze the visual world. However, the neurons in the early visual areas rarely receive such simple patterns as those that have been extensively used in vision research. In addition, most of these neurons mainly exhibit nonlinear behavior in the presence of more complex inputs. As a result, studying vision under naturalistic conditions is revealed to be an essential step to confirm the models obtained from the studies with simple stimuli. Specifically in the domain of eye movement research, there has been over a century of tradition in studying saccadic eye movements using simple visual stimuli such as light flashes, lines, and Gabor patches [34, 35]. Nevertheless, the extension of such findings to the more realistic visual stimuli is questionable. Moreover, recent technological improvements enable the modification of visual stimuli in real-time, depending on the viewers' gaze direction, which is known as the gaze-contingency paradigm. Using this paradigm, it is now possible to study naturally generated saccades with real-life stimuli. For example, in a recent study providing evidence against the extraretinal source of saccadic suppression, a gaze-contingent high-definition display was developed to modify videos in retinal coordinates in real-time [14]. The present work is one of the few examples of extending the validity of saccadic suppression to natural complex objects.
